# The Use of Raman Spectroscopy to Monitor Metabolic Changes in Stressed *Metschnikowia* sp. Yeasts

**DOI:** 10.3390/microorganisms9020277

**Published:** 2021-01-29

**Authors:** Andrea Němcová, Dominika Gonová, Ota Samek, Matthias Sipiczki, Emilia Breierová, Ivana Márová

**Affiliations:** 1 Faculty of Chemistry, Brno University of Technology, Purkyňova 464/118, 612 00 Brno, Czech Republic; xcgonova@fch.vut.cz (D.G.); marova@fch.vut.cz (I.M.); 2 Institute of Scientific Instruments of the Czech Academy of Sciences, Královopolská 147, 612 64 Brno, Czech Republic; osamek@isibrno.cz; 3 Department of Genetics and Applied Microbiology, Faculty of Science and Technology, University of Debrecen, Egyetem tér 1, 4032 Debrecen, Hungary; lipovy@gmx.com; 4 Institute of Chemistry, Slovak Academy of Sciences, Dúbravská Cesta 9, 845 38 Bratislava, Slovakia; emilia.breierova@savba.sk

**Keywords:** yeasts, *Metschnikowia*, lipids, pulcherrimin, Raman spectroscopy

## Abstract

Raman spectroscopy is a universal method designed for the analysis of a wide range of physical, chemical and biological systems or various surfaces. This technique is suitable to monitor various components of cells, tissues or microorganisms. The advantages include very fast non-contact and non-destructive analysis and no or minimal need for sample treatment. The yeasts *Metschnikowia* can be considered as industrially usable producers of pulcherrimin or single-cell lipids, depending on cultivation conditions and external stress. In the present study, Raman spectroscopy was used as an effective tool to identify both pulcherrimin and lipids in single yeast cells. The analysis of pulcherrimin is very demanding; so far, there is no optimal procedure to analyze or identify this pigment. Based on results, the strong dependence of pulcherrimin production on the ferric ion concentration was found with the highest yield in media containing 0.1 g/L iron. Further, production of lipids in *Metschnikowia* cells was studied at different temperatures and C:N ratios, using Raman spectroscopy to follow fatty acids composition, under different regimes, by monitoring the iodine number. The results of Raman spectroscopy were comparable with the fatty acid analysis obtained by gas chromatography. This study therefore supported use of Raman spectroscopy for biotechnological applications as a simple tool in the identification and analysis both the pulcherrimin and microbial lipids. This method provides a quick and relatively accurate estimation of targeted metabolites with minimal sample modification and allows to monitor metabolic changes over time of cultivation.

## 1. Introduction

Raman spectroscopy is a spectroscopic technique with many types of applications; it is commonly used to provide a structural fingerprint and composition of substances [1,2]. The method is based on inelastic scattering of photons, known as Raman scattering, that allows identification of exact molecular spectrum of biological/non-biological sample [3,4,5,6]. Conventional analytical techniques, such as gas chromatography or high-performance liquid chromatography, are commonly used to detect different metabolites or lipids [7,8]. However, these conventional technologies are destructive, time-consuming and often expensive. Spectroscopic techniques, also including Raman spectroscopy, have gained importance for rapid, non-destructive and inexpensive detection [9,10].

All types of cells may be characterized by the ability to respond to changes in their environment [3,5,11]. The main goal of our investigations is the development of specialized instrumentation and methodology to monitor metabolic states of microorganisms in order to optimize cultivation process for biotechnological applications, using Raman spectroscopy. Production properties of yeast strains (from genus *Metschnikowia*) were tested by Raman spectroscopy. It can be exploited in instances where fast and accurate monitoring/determination of samples is required [1,6].

Yeasts of the *Metschnikowia* genus are relatively widespread in nature. More than 35 species of this yeast genus have been defined. These have been isolated from various sources, such as flowers and fruits or from the bodies of invertebrates, insects or even human skin. Some species also occur freely in nature, based on which they can be divided into aquatic species and terrestrial species. The potential for the use of *Metschnikowia* genus yeasts is relatively high. Some species of this genus are characterized by their antimicrobial activity, making them suitable in agricultural use, mainly as a prevention against post-harvest diseases in fruit, e.g., *M. pulcherrima* or *M. fructicola*. Some of the *Metschnikowia* yeasts can also produce industrially important substances, such as acid protease (*M. reukaufii*) [11,12,13].

The interest in these yeast species in the biotechnology industry is mainly conditioned by their ability to synthesize a pigment called pulcherrimin. Pulcherrimin has antimicrobial effects against bacteria, but especially yeasts and fungi. This makes yeasts of this species very attractive for use as a biocontrol agent. Pulcherrimin is a ferric salt of the dibasic pulcherriminic acid (3,6-dihydroxy-2,5-diisobutylpyrazine-1,4-dioxide). The complex structure of pulcherrimin is formed by an iron chelate in which the four oxygen atoms from pulcherriminic acid are coordinated with two iron atoms. It occurs as a red extracellular pigment formed by several bacteria species and some yeasts after growth in media enriched in iron (III). Thus, the antagonistic activity of pulcherrimin is likely to be closely related to the depletion of ferric ions from the nutrient medium required for its synthesis by the cell, thereby competing for a common source with potential pathogens. The production of pulcherrimin is dependent on the presence of oxygen and significantly affected by the amount of free iron in the medium [14,15,16,17,18].

Pulcherrimin is very slightly soluble in water, probably only in the colloidal phase and insoluble in all organic solvents. On the contrary, it is easily soluble in strong alkaline solvents. It is unusually resistant to relatively strong acids. Degradation of pulcherrimin occurs at approximately 150 °C [14,16,19]. The analysis of pulcherrimin is very difficult, due to its chemical properties and structure. Usually its identification is performed only by visual observation of yeast colonies or their environment. In the past, the pigment was converted to its precursor, pulcherrimic acid, and subsequently identified by infrared spectroscopy. In this work, we have attempted to identify pulcherrimin and pigments formed in the presence of other metals, using Raman spectroscopy [17,18,19,20,21].

In addition to antimicrobial activity, several studies in recent years have focused on the possibilities of lipid production and regulation by yeasts of the genus *Metschnikowia* [22]. Lipids produced by microorganisms (oleogenic microorganisms) are referred to as “single-cell oils”. An important ability of these microorganisms is using waste substrates from various industrial productions for lipid production. Especially from the food and agricultural industry. In addition to the automotive industry, microbial lipid production is of interest, due to the ability of microorganisms to synthesize lipids that contain polyunsaturated fatty acids. These fatty acids are of great importance in the medical and dietary industry, or lipids whose composition is like those contained in cocoa butter. In addition, microbial lipids can be used as a source of lipids in the manufacturing process of detergents, soaps, plastics, paints and additives for the cosmetic industry [20,23,24,25,26,27].

The presented work deals with the screening of various types of culture conditions for lipid production by selected yeast species of the genus *Metschnikowia*, too. Raman spectroscopy was used for the initial estimation of lipid production, depending on the culture conditions. This method is a fast and efficient tool compared to conventional techniques.

## 2. Materials and Methods

### 2.1. Strains and Culture Conditions

For the monitoring of pulcherrimin production abilities, four *Metschnikowia* yeast strains have been selected: *Metschnikowia pulcherrima* 29-02-145, *Metschnikowia pulcherrima* 29-02-147, *Metschnikowia pulcherrima* 29-02-149 and *Metschnikowia andauensis* 29-02-129. These strains were purchased from the Culture Collection of Yeast (CCY), Institute of chemistry, Slovak Academy of Science, Bratislava, Slovakia.

Yeast strains were cultivated by using pre-step inoculation. The cultivation of the inoculum was performed in Erlenmeyer flasks. The inoculum was cultivated for 24 h at 25 °C, under continuous shaking. Inoculum was then transferred into production medium (volume ratio 1:5) and grown under the same conditions.

The yeast strains were grown on medium (inoculum/production medium) which is composed of (g/L): glucose 20; bacteriological peptone 5; yeast extract 10; and salts—KH_2_PO_4_ 1; K_2_HPO_4_ 0.2; NaCl 0.1; CaCl_2_ 0.1; MgSO_4_ 0.5; agar 20; and maintained at 4 °C.

For monitoring of lipid production abilities, some other *Metschnikowia* yeast strains have been selected in addition to the abovementioned: *Metschnikowia chrysoperlae* CBS 9803 (11-1158), *Metschnikowia pulcherrima* CBS 5833 (11-1232), *Metschnikowia fructicola* CBS 8853 (11-1235), *Metschnikowia andauensis* HA 1657 (11-1241) and *Metschnikowia sinensis* CBS 10357 (11-1244). These strains were purchased from Centraalbureau voor Schimmelcultures, Delft, (CBS), Netherlands.

The ability of these yeast strains to accumulate lipids was observed depending on different culture and stress conditions, in particular the ratio of carbon to nitrogen source in the medium at low temperature 15 °C.

### 2.2. Revitalization of Yeast Strains

Two biological replicates were prepared for each strain and cultivation medium by inoculating strains from frozen cryopreserved stocks onto agar plates.

### 2.3. Screening of C/N Ratio

Cultures were prepared to maintain a selected C/N ratio of 24, 97 and 150, while using glucose as a carbon source. For the resulting C/N, the amount of glucose in the individual media varied in proportion to the bacteriological peptone and yeast extract. The medium with (i) C/N ratio 24: glucose 20 (g/L), bacteriological peptone 5 (g/L) and yeast extract 10 (g/L) and salts (see Section 2.1); (ii) C/N ratio 97: glucose 30 (g/L), bacteriological peptone 0.68 (g/L) and yeast extract 0.34 (g/L) and salts; (iii) C/N ratio 150: glucose 30 (g/L), bacteriological peptone 0.34 (g/L) and yeast extract 0.34 (g/L) and salts.

The experiments were carried out in 250 mL Erlenmeyer flasks sterilized at 121 °C/ 25 min. All flasks were cultivated at 25 °C for 3 days and then at 15 °C for 11 days, under shaking. Control series were cultivated at 25 °C for 14 days. All experiments were carried out in triplicate. Cultivation volume of the media was set to 50 mL.

### 2.4. Cell Preparation

Yeast biomass (10 mL culture) were centrifuged at 5000 rpm g, for 5 min, to spin down the yeasts. The supernatant was collected and discarded. Then, the yeast cells were washed twice with a mixture of distilled water and hexane 1:1 (*v/v*) and resuspended in 1 mL of distilled water. Then, the washed biomass was quantitively transferred into Eppendorf tubes, frozen at −20 °C and then freeze-dried. After determining their weight, to calculate cell dry weight (CDW), the dried cells were used for the analysis of lipids by gas chromatography.

### 2.5. Gas Chromatography Analysis

The total lipids and individual fatty acids were determined by optimized gas chromatography/flame ionization detection (GC/FID) analysis. Lipids were extracted from 10 mg of freeze-dried cells by the Folch method (methanol:chloroform, 2:1) [28] and converted into their fatty acid methyl esters (FAMEs). The FAMEs were analyzed by gas chromatography (GC) analysis. GC analysis of FAMEs was carried out on a TRACETM 1300 Gas Chromatograph (Thermo Fischer Scientific, Waltham, MA USA) equipped with an Al 1310 autosampler, a flame ionization detector and a Zebron ZB-FAME column, where the bleed specification at 260 °C was 3 pA (30 m, 0.25 mm, 0.20 µm). Fatty acids were identified by comparison to commercial FAME standards (FAME37, Supelco, Sigma, St. Louis, MO, USA) and quantified by the internal standard method, involving the addition of 100 µg of commercial heptadecanoic acid (Sigma-Aldrich, Merck, Steinheim, Germany). Chromatography data were evaluated by Chromeleon software 7.2 (Thermo Fischer Scientific, Waltham, MA, USA). The total lipid concentration, based on GC data, was evaluated, as well [29].

### 2.6. Raman Analysis

To prepare samples for Raman analysis, the following steps were performed: 1 mL of yeast culture was transferred from the cooling box into 1.5 mL tube and the cells were centrifuged (10,000 rpm, 2 min). In the next step approximately 20 µL of cell pellets formed by centrifugation was pipetted onto a CaF (Raman grade) microscope slide. The cell suspensions were analyzed, using Raman microspectrometer.

The yeast suspension was analyzed by using the Renishaw inVia system (Renishaw inVia Raman Spectrometer, Renishaw plc., Wotton-under-Edge, UK), with 785 nm single-mode diode laser as the excitation source. The laser beam was focused onto a sample by the microscope objective (Leica, Wetzlar, Germany, 50×, NA (Numerical aperture) 0.5) with the laser spot diameter of approximately 2 μm × 10 μm (note that such laser spot shape is characteristic for the Renishaw inVia instrument), with full axial depth of the excitation region at 8 µm. The laser was focused onto a surface of the sample. Overview spectra were acquired in the range of 700–1800 cm^−1^. Each spectrum was measured for 15 s.

### 2.7. Raman Data Processing

The Raman spectra were treated with the Savitzky–Golay procedure coupled with advanced rolling filter background removal routine (using the program written in-house, using MatLab software (MathWorks, Natick, MA, USA)) [30,31].

### 2.8. Statistical Analysis

The growth experiments in Erlenmeyer flasks were carried out in triplicate. The presented results are the mean of the replicates from cultivation and GC analysis, and the standard deviations are shown in the tables. Statistics were performed by using the Excel software package (Microsoft Excel 2013, Microsoft Corp., Redmond, WA, USA).

## 3. Results and Discussion

### 3.1. Monitoring of Production of Pulcherrimin

Pigment pulcherrimin depletes the free iron from the environment or iron present in the media. The pulcherrimin pigment has brown-red color—as seen on the colonies—and is produced only when the cells are grown in the presence of iron. Pulcherrimin has antimicrobial activity against pathogens, microscopic fungi and other yeast species. Moreover, pulcherrimin potency to exert antagonistic effects of *Metschnikowia* yeasts correlates with the amount of pulcherrimin production. The antimicrobial activity occurs when iron immobilizing pulcherrimin, together with pulcherriminic acid, sequesters any iron in the environment which, in turn, prevents other microorganisms’ growth, because iron is an element vital for the growth. Thus, because of this remarkable features pulcherrimin plays important role in ecosystem and is of great interest for different biotechnological applications [22,32].

In the following, we aim to explore pulcherrimin production characteristics on selected samples when yeast is cultivated in the presence of different concentrations of ferric ions.

On the assumption that pulcherrimin is formed by chelation of pulcherriminic acid and iron ions we can consider that chelation is not limited only on iron ions and should appear also for different metal ions. Thus, adding, e.g., copper ions to media pigments could be produced—this was experimentally tested in our experiments.

The synthesis of pulcherrimin is only possible if the oxygen is supplied. Consequently, in an anaerobic environment pulcherrimin cannot be secreted. The production of pulcherrimin depends on many factors, e.g., small changes in pH or oxidative stress.

Identification of pulcherrimin and other pigments produced in the presence of iron or copper was monitored by visual observation directly on Petri dishes and with Raman spectroscopy.

#### 3.1.1. Screening and Identification of Pulcherrimin Production in the Presence of Iron Ions by Visual Observation and by Raman Spectroscopy

For this experiment, selected yeast species *Metschnikowia pulcherrima 145, Metschnikowia pulcherrima 147, Metschnikowia pulcherrima 149* and *Metschnikowia andauensis 129* capable of producing pulcherrimin were used. The composition of the individual media used and the culture conditions are given in Section 2.1. In all the abovementioned yeast cultures of the genus *Metschnikowia*, color changes were visually observed depending on the medium used, and in some selected strains, the identification of pulcherrimin was also performed by Raman spectroscopy, in order to present the capability of this method for pulcherrimin detection.

For monitoring by visual observation, the method of agar media supplemented with ferric ions directly on Petri dishes was used. Consequently, changes in color intensities were monitored depending on produced pulcherrimin.

The cultivation media differed in concentration of Fe ions. The composition of media was as stated in Section 2.1 with addition of metal compound, iron in the form of FeCl_3_ (0.1 g/L, 0.01 g/L or 0.05 g/L).

**Figure 1:**
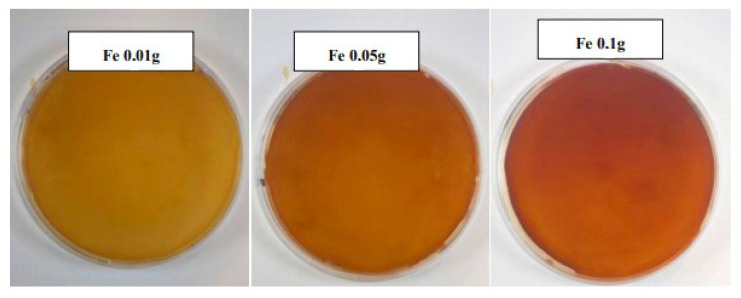
Color changes in *Metschnikowia pulcherrima* 147. Indicated amounts of ferric-ions were added to the media, which are accompanied by the media color change.

By adding ferric ions to the medium, pulcherrimin production is observed from pale brown to reddish brown. The intensity of color staining increased with the increasing iron concentration. From the color saturation of the media, the highest pulcherrimin production can be expected in media containing 0.1 g/L iron (Figure 1).

Pulcherrimin production by various yeast and bacteria increases when free iron oxide ions are present in cultivation media. There in an iron concentration level which determines whether pigment is intracellular or extracellular. If the composition of iron in media is at the certain level, pulcherrimin pigmentation can be found in biomass and also in media. This level is defined as critical level and differs for different bacterial and yeast strains. In case the iron level is below critical level, pigment is found in media, when the iron level is higher in media pigmentation occurs in biomass and media does not change color. Thus, biomass or media color intensity depends on concentration of iron ions.

The colors of the colonies changed from white to dark red with increasing iron concentration. Stains began to appear around colonies of cells diffused into the agar medium. The red color of the cells was an indication that a reddish-brown pigment is formed by yeast cells. Due to the diffusion of the precursor into agar and the reaction with iron (III), various precipitated red patterns could be obtained.

The presence of pulcherrimin produced by *Metschnikowia* cells cultivated in media with different iron (Fe) concentration was detected based on the intensity ratio of the specific Raman peaks found for pulcherrimin at 1405 and 1435 cm^−1^.

**Figure 2:**
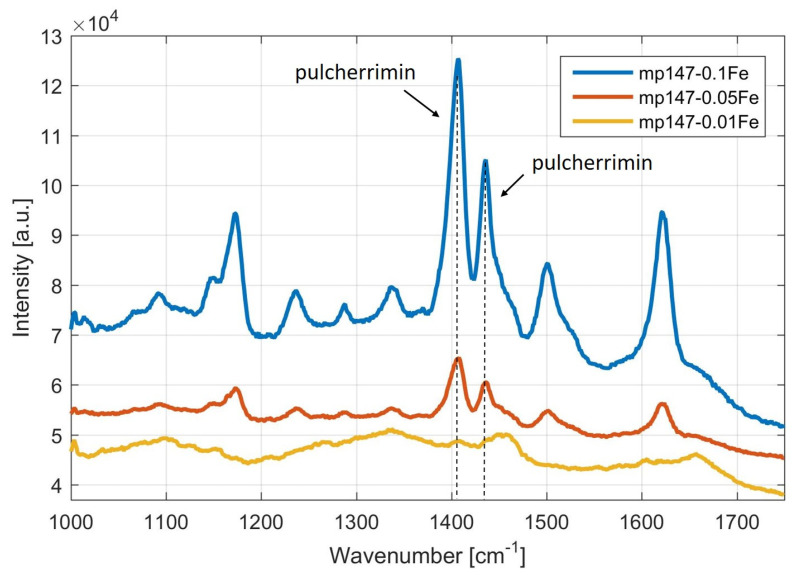
Raman spectra of pulcherrimin produced by yeast *Metschnikowia pulcherrima* MP 147 on three media with different iron concentrations. Legend: *Metschnikowia pulcherrima* 147 cultivated on medium with 0.01 g/L Fe (mp147—0.01Fe, yellow trace), medium with 0.05 g/L Fe (mp147—0.05Fe, red trace) and medium with 0.1 g/L Fe (mp147—0.1Fe, blue trace). The two main Raman spectral lines of pulcherrimin can be found at 1405 and 1435 cm^−1^ [33,34]. Spectra are vertically shifted for clarity.

As it can be seen (Figure 2), in the Raman spectra of pulcherrimin produced by *Metschnikowia pulcherrima* 147, in media with different iron concentrations, the strong dependence of pulcherrimin production on the ferric ion concentration is obvious. Estimating from visual observation of changes in the color of these media, detection by Raman spectroscopy confirmed that the highest pulcherrimin production was achieved in media containing 0.1 g/L iron.

**Figure 3:**
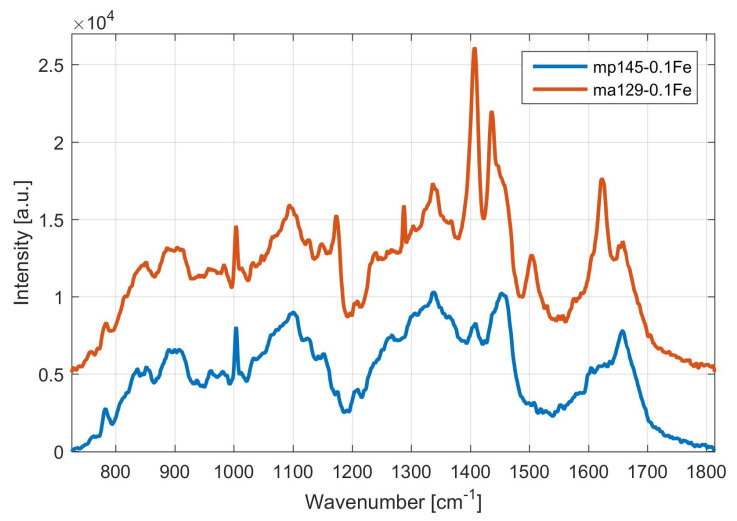
Raman spectra of pulcherrimin produced in media containing 0.1 g/L Fe by yeast *Metschnikowia pulcherrima* MP145 and *Metschnikowia andauensis* MA129. Legend: *Metschnikowia andauensis* 129 cultivated on medium with 0.1 g/L Fe (ma129-0.1Fe, red trace) and *Metschnikowia pulcherrima* 145 cultivated on medium with 0.1 g/L Fe (mp145—0.1Fe, blue trace). Spectra are vertically shifted for clarity.

In Figure 3, which depicts the Raman spectra, the concentration of pulcherrimin produced by different yeasts—*Metschnikowia pulcherrima* 145 and *Metschnikowia andauensis* 129—in the same media containing 0.1 g/L Fe was compared. Visibly higher concentrations of pulcherrimin (peak at 1405 cm^−1^) can be observed in yeast *Metschnikowia andauensis* 129. This strain is likely to produce a larger amount of precursor, pulcherriminic acid, which, in the presence of ferric ions, becomes pulcherrimin. *M. andauensis* 129 strain, due to the higher production capabilities of the pigment, may have a higher antifungal activity, may inhibit fungal growth on certain types of food and thus be used for scale-up production of this bio-protection agent.

The period of cultivation and the iron concentration in the medium were very important parameters for the color intensity.

Yeast producing large amounts of pulcherrimin in the presence of ferric ions is in the focus for different biotechnological applications as potential inhibitor of pathogenic microorganisms. We showed that a larger amount of pigment, which correlates with higher antifungal activity, can be monitored by visual observation and also with Raman spectroscopy. Thus, Raman spectroscopy can be used as a tool for fast pulcherrimin monitoring in applied biotechnology.

#### 3.1.2. Monitoring of Pulcherrimin Production Depending on the Presence of Other Metal Ions in the Medium Visually and by Raman Spectroscopy

For this experiment, yeast *Metschnikowia pulcherrima 145, Metschnikowia pulcherrima 147, Metschnikowia pulcherrima 149* and *Metschnikowia andauensis 129* capable of producing pulcherrimin were used. The composition of the individual media used and the culture conditions are given in Section 2.1.

The cultivation media differed in concentration of Cu ions in the form of CuSO_4_ (0.05 g/L).

**Figure 4:**
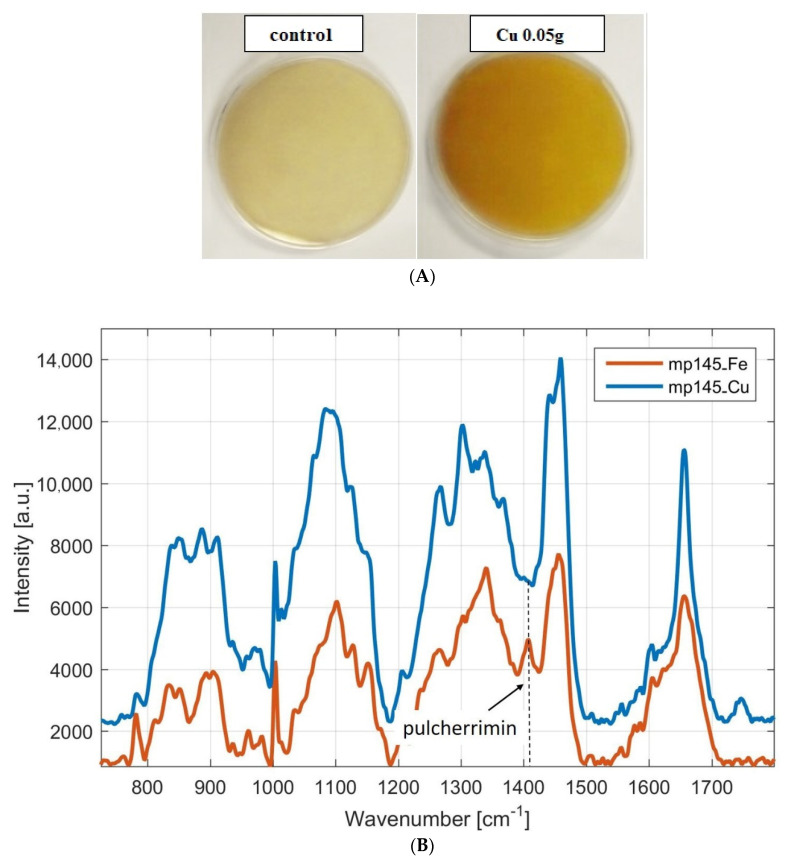
(**A**) Color changes in *Metschnikowia pulcherrima* 145. Copper-ions were added to the media which is characterized by the media dark yellow color change (CuSO_4_ 0.05 g/L). Control is medium without metal ions. (**B**) Raman spectra of pulcherrimin produced by yeast *Metschnikowia pulcherrima* MP 145 on Cu-containing medium and medium with iron ions. Legend: *Metschnikowia pulcherrima* 145 cultivation on medium with 0.05 g/L Fe (mp145_Fe, red trace) and medium with 0.05 g/L Cu (mp145_Cu, blue trace). Spectra are vertically shifted for clarity.

The coloring of the individual media, depending on the composition, was analogous in all strains. In all four strains, the control medium was pale yellow, indicating that in these media yeasts did not produce pulcherrimin, which is typical of its brown color. The presence of copper in the medium caused a discoloration of the stains around the cell colony. Copper-ion-containing media were dark yellow in color, compared to the control, suggesting that a pigment with chelating properties was produced, but this was not pulcherrimin (Figure 4A).

The Raman spectra shown in the Figure 4B demonstrate the production of pulcherrimin by *Metschnikowia pulcherrima* 145 on two different media—medium containing copper (CuSO_4_ 0.05 g/L) and medium with containing iron (FeCl_3_ 0.05 g/L). As could be expected from visual observations, pulcherrimin production is not observed in the Raman spectrum when copper is added to the medium (Raman peak found at 1405 cm^−1^). Vice versa, the Raman spectrum of yeast grown in iron-containing medium shows pulcherrimin production. It is therefore likely that the resulting dark yellow pigment with chelating properties will not have an antifungal effect of pulcherrimin.

Further, we will follow on our experiments and discuss possibilities of pulcherrimin production, using different composition of nutrient media. Four yeast strains were used as the micro-organisms synthesizing pulcherrimin: *Metschnikowia pulcherrima* 145, *Metschnikowia pulcherrima* 147, *Metschnikowia pulcherrima* 149 and *Metschnikowia andauensis* 129. The media differed in the concentrations of metal ions; specifically, iron in the form of FeCl_3_ (0.1 g/L, 0.01 g/L or 0.05 g/L) (you can see at Section 3.1.1) or copper in the form of CuSO_4_ (0.05 g/L) were applied (you can see at Section 3.1.2). Subsequently, visual changes in color were observed, and the pigment was analyzed by Raman spectroscopy. Based on the results, it was shown that all strains of the genus *Metschnikowia* could produce pulcherrimin, but only on nutrient media enriched with ferric ions. As expected from visual observation, in the case of the addition of copper to the medium, no production of pulcherrimin is observed in the Raman spectrum (peak around 1405 and 1435 cm^−1^). As iron concentration increased, unequivocally increased staining intensity was observed, suggesting increased pigment production. This assumption was also confirmed by Raman spectroscopy, where the highest pulcherrimin production was recorded in a medium containing 0.1 g/L ferric ions, namely for *Metschnikowia pulcherrima* 147 (Figure 1 and Figure 2). We have found that the data for strain *Metschnikowia pulcherrima* 149 are very similar to other strains, and this is why we do not present the details. Analysis of pulcherrimin production by Raman spectroscopy is a very efficient and fast tool. The monitoring takes only few minutes. In the past, J.C. Macdonald, in cooperation with W. C. Haid, analyzed pulcherrimin and its precursor pulcherrimic acid by infrared spectroscopy [15,33]. However, the analysis was preceded by time-intensive and manually intensive isolation [18]. In 2020, shell-isolated nanoparticle-enhanced Raman spectroscopy was successfully applied for characterization of pulcherrimin [34]. Advances in genus *Metschnikowia* have been illustrated in two recent publications [35,36].

### 3.2. Rapid Monitoring of Lipid Production by Metschnikowia Yeasts, Using Raman Spectroscopy

In the study of lipid production by the yeast genus *Metschnikowia*, changes in the amount of saturated and unsaturated fatty acids during each cultivation were observed by using Raman spectroscopy as well. Specific peaks in the Raman spectra correspond to saturated and unsaturated carbon bonds, which allows a rapid estimation of the degree of lipid unsaturation, iodine number, based on the intensity ratio of specific peaks for saturated fatty acids (FA) (CH_2_; 1445 cm^−1^) and unsaturated FA (C=C; 1656 cm^−1^) [31].

**Figure 5:**
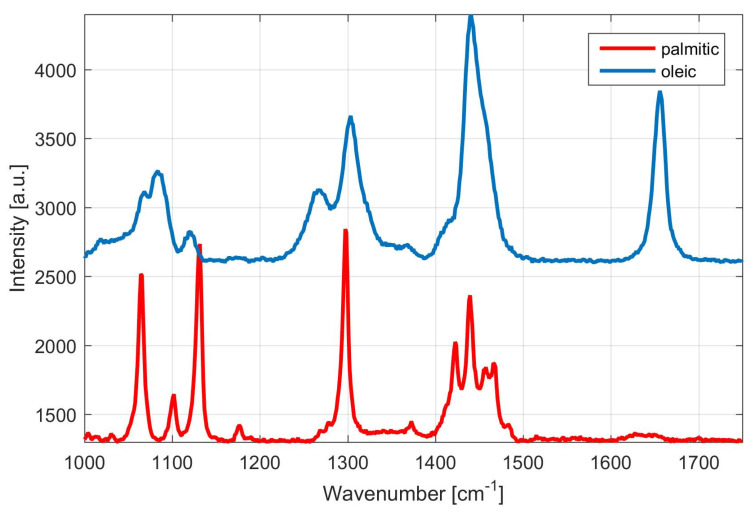
Typical Raman spectrum of palmitic (bottom trace) and oleic acid (upper trace). Spectra are vertically shifted for clarity. Graph illustrates differences between monounsaturated fatty acid (oleic) and saturated fatty acid (palmitic).

**Figure 6:**
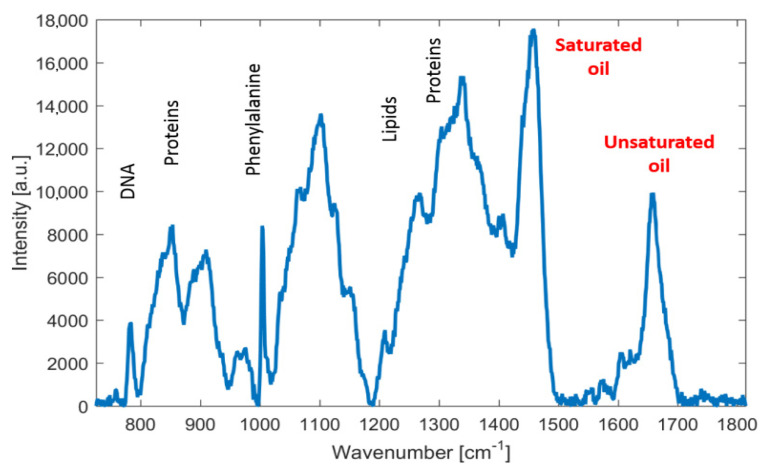
Raman spectra of oil fraction extracted from *Metschnikowia pulcherrima* 29-2-149. Selected emission lines are highlighted - the two lines used for the iodine number estimation are marked in red.

In order to demonstrate fatty acids that predominate in our samples, a typical Raman spectrum of palmitic (bottom trace) and linoleic acid (upper trace) is shown in Figure 5. These derivatives represent main composition of fatty acids (saturated and unsaturated) in a drop of palm oil—palmitic oil of about 45% (saturated) and linoleic acid (unsaturated) of about 10%. Consequently, Figure 6 shows Raman spectrum of an oil drop extracted from centrifuged suspension of *M. pulcherrima* 149, which is composed of these predominating fatty acids. Thus, Raman spectroscopy is a potentially important tool for monitoring fatty acids composition (on-line and in near-real time) during the cultivation under different temperatures regimes simply by monitoring the iodine number [31] of the given sample.

#### Monitoring of Fatty Acid Production Changes during Cultivation, Using Raman Spectroscopy

**Figure 7:**
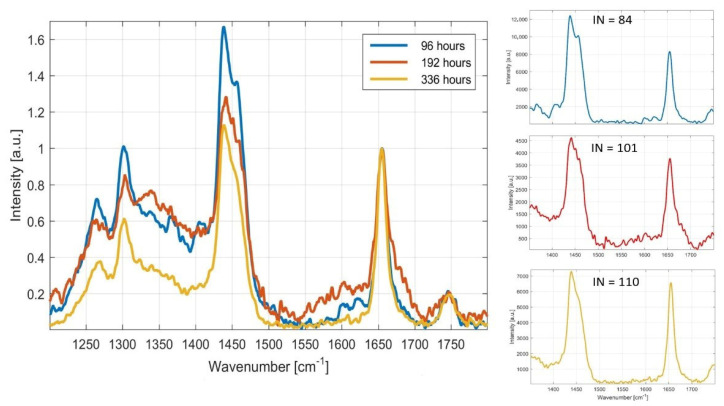
Changes of unsaturation of FA during the cultivation of *M. pulcherrima 149* for the C/N ratio 97. Legend: (1) 96 h of cultivation (96 h, blue trace), (2) 192 h of cultivation (192 h, red trace) and (3) 336 h of cultivation (336 h, yellow trace). The main spectra are normalized for clarity (peak at 1656 cm^−1^). Three insets on the right show details for each spectrum, so that intensities and, in turn, ratios of both peaks can be compared. Calculated iodine numbers (INs) are indicated according to the equation at Figure 10. Figure shows that, with increasing cultivation time, unsaturation of fatty acids is also increased.

Raman spectra of *M. pulcherrima* 149 for the C /N ratio 97 cultivated for different time period of 96; 192 and 336 h are presented in the Figure 7. Changes of unsaturation of FA based on the ratio of specific peaks of unsaturated fatty acids and saturated fatty acids were recorded. An increase in unsaturated FA (peak 1656 cm^−1^) can be observed with increasing cultivation time.

**Figure 8:**
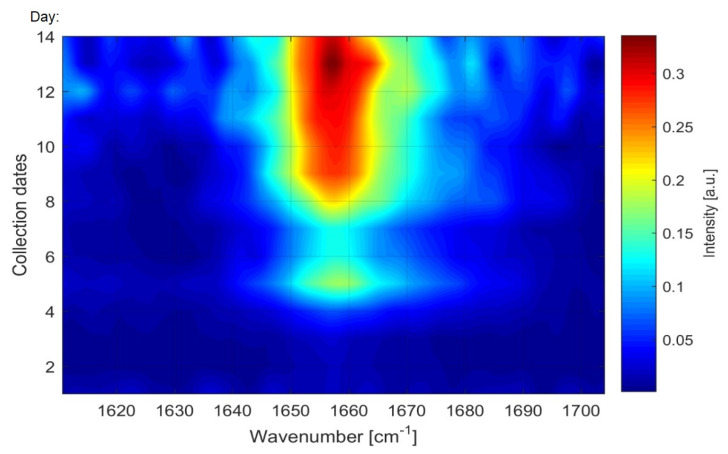
Spectral map plotted using Raman line intensities for unsaturated oil for 14 collection dates (days of cultivation). The map qualitatively monitors unsaturated oil within the cells (*Metschnikowia andauensis* 129) at different collection dates. Higher intensity refers to higher levels of unsaturation.

In addition, spectral maps can be used to visualize the time changes of saturated and unsaturated FA detected by Raman spectroscopy, which clearly outlines the course of increasing unsaturated FA with increasing cultivation time (Figure 8).

From the spectral map, very low levels of unsaturated fatty acids can be observed at the beginning of cultivation and their gradual increase over time, as shown by a color intensity scale. The results obtained with Raman spectrometry indicate the effect of cultivation time on the presence of saturated and unsaturated FA in the lipids of yeasts cultured on glucose. A longer cultivation time stimulates the formation of unsaturated FA in microbial lipids accumulated in *Metschnikowia* cells.

Table 1 shows the fatty acid content on selected days of cultivation obtained by gas chromatography. The results are consistent with Raman spectra and correspond to a gradual increase in unsaturated fatty acids with increasing cultivation time. Raman spectroscopy, therefore, proved to be a very efficient tool for the rapid quantitative/qualitative analyses of oil produced by yeast. This may take a few minutes, compared to conventional instrumentations (such as GC).

### 3.3. Analysis of Lipid Production and Fatty Acid Distribution at Different C/N Ratio

The biomass production and lipid accumulation in oleaginous yeasts are mainly affected by the C/N ratio and temperature regime of medium [37,38]. The effect of the C/N ratio at low temperature on the production of biomass and lipid accumulation was studied in all tested *Metschnikowia* strains, and the results are shown in Table 2. For the production of biomass (g/L), the percentual content of fatty acids per biomass weight (%) was monitored in each cultivation (glucose as a C source), at a different C/N ratio.

Most of the used strains resulted in higher biomass production when cultivated on the glucose medium with low C/N ratio (24 or 97). An exception is *M. chrysoperlae* 1158, which has a biomass yield for both C/N ratios, was similar (C/N 97: 6.9 ± 0.7 g/L and C/N 150: 7.1 ± 0.5 g/L). Cultivations with an increased C/N ratio caused higher variability in the lipid production in individual strains, as well as among different media. *M. pulcherrima* 149 yeast showed a more significant lipid overproduction (8.4%) on the glucose medium with C/N ratio 150, from strains obtained from the Culture Collection of Yeast (CCY). Strains which were obtained from Centraalbureau voor Schimmelcultures, Delft, (CBS) are better lipids producers. *Metschnikowia andauensis* 1241 was one of the best lipid producers, based on our experiments (19.6 ± 2.5% lipid content from DCW).

At the same time, these higher C/N ratios were accompanied with the highest production of lipids by all used yeast strains. Likewise, Canonico et al. achieved the maximum yield at a higher C/N ratio, namely the ratio C/N 118, which is in the range of C/N ratios (97 and 150) examined in this work, which proved to be more suitable for inducing microbial lipid production [38].

In a more detailed analysis, biomass and lipid production were monitored at C/N ratios of 97 and 150. By culturing yeast on glucose media with C/N 97 and C/N 150 ratio under reduced temperature conditions (15 °C), relatively high lipid production was achieved. Same as for the culture medium with a C/N ratio of 97, and also with C/N ratio of 150, the producer with the highest lipid content was *M. andauensis* 1241. When comparing biomass or yeast lipid productivity at different C/N ratios, although lipid production was generally higher at C/N 150, more biomass was recorded at a C/N ratio of 97. This phenomenon could be due to lower nitrogen content source, to its faster depletion and accumulation of lipids. In principle, however, there was no significant variation in lipid or biomass production on both culture media.

**Figure 9:**
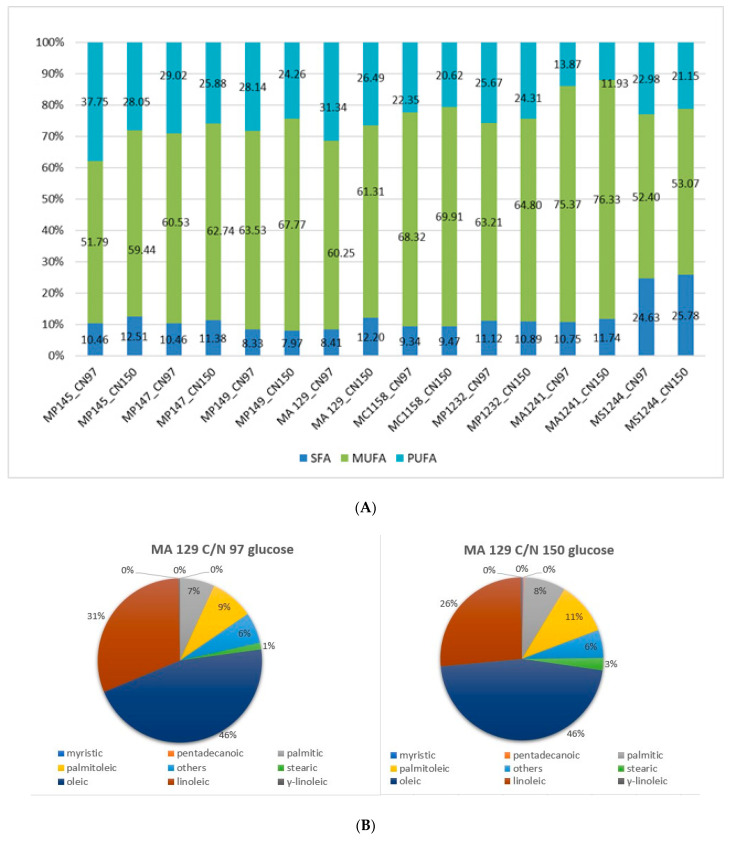

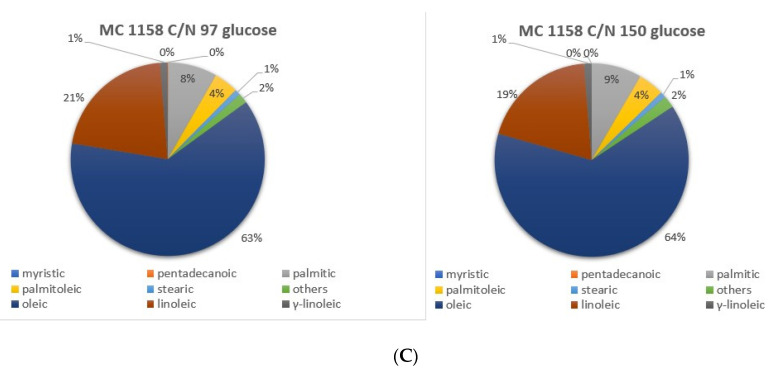
(**A**) Percentage of saturated (SFA), monounsaturated (MUFA) and polyunsaturated (PUFA) FA in selected yeast strains grown on glucose medium with C/N ratio of 97 and 150. (**B**) Percentage of selected fatty acids in *Metschnikowia andauensis* 129 grown on glucose medium with C/N ratio of 97 and 150. (**C**) Percentage of selected fatty acids in *Metschnikowia chrysoperlae* 1158 grown on glucose medium with C/N ratio of 97 and 150.

From the analysis of fatty acids by gas chromatography (Figure 9A), lipids produced by *M. pulcherrima* yeasts were composed mostly from monounsaturated fatty acids in all media used, with a higher C/N ratio. It was most significant for *Metschnikowia chrysoperlae* 1158 (C/N 97: 68.32 and C/N 150: 69.91% MUFA) and *Metschnikowia andauensis* 1241 (C/N 97: 75.37 and C/N 150: 76.33% MUFA). The use of glucose carbon substrate significantly affected the presence of fatty acid groups; on the other hand, the ratio of carbon and nitrogen in the culture medium had only a slight effect on the FA composition.

The major fatty acids produced by *Metschnikowia* yeasts cultivated on glucose medium with the same C/N ratio were oleic acid and linoleic acid. Oleic acid is present in about 50%. In the Figure 9B,C, a lower or similar proportion of monounsaturated fatty acids in the glucose medium with C/N ratio 97 can be seen, but on the other hand, also a higher amount of polyunsaturated fatty acids, in comparison with C/N ratio of 150.

The presence of linoleic acid in larger amounts was also again observed in all strains. However, the trend of linoleic acid accumulation was the opposite of glucose media with C/N 97 and 150, when compared to oleic acid. By increasing the C/N to 150, a decrease in linoleic acid accumulation was observed in all strains.

A significant proportion of saturated fatty acids were composed by palmitic and stearic acids. The same character of the fatty acid profile was also observed in the other studied *Metschnikowia* yeasts.

In further experiments, *Metschnikowia sinensis* 1244 was cultivated in media containing both C/N ratios. We monitored production of palmitic and stearic acids, too. Production of stearic acid (18:0) was at 13.48% the highest amount from strains covered by our study. The other strains under our investigations produced only about 2% of stearic acid.

Fatty acid profiles in lipids produced by *Metschnikowia* strains which were analyzed in our study can be broadly compared with the study of Santamauro et al. (2014). In our experiments, the production of linoleic acid was higher, and this can be attributed to the C/N ratio; this was different from the study by Santamauro et al. [37].

**Figure 10:**
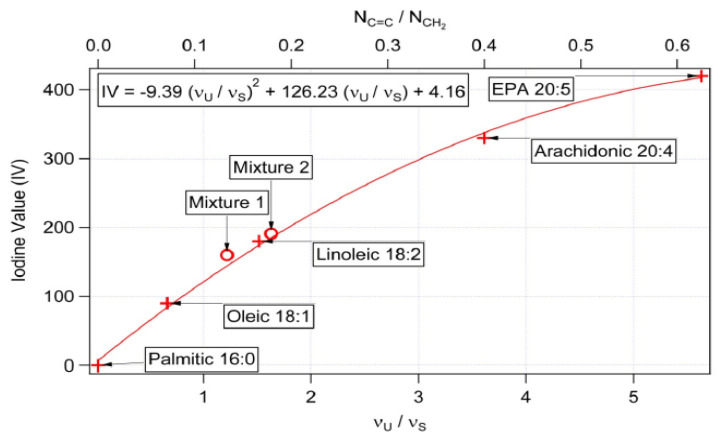
Calibration curve for iodine number determination [31].

Raman spectroscopy serves as an efficient and easy alternative method also for lipid detection, as demonstrated above. This technique provides a quick detection of the saturated and unsaturated bonds of lipids contained in yeast cells. The intensity of specific spectral peaks can be used for the estimation of unsaturation degree in the form of the iodine number [31]. The spectral peaks νU at 1656 cm^−1^ (proportional to the amount of unsaturated C = C bonds) and νS at 1445 cm^−1^ (proportional to the amount of saturated C-C bonds) were used [31]. The iodine number of each sample was determined by using the equation derived in Figure 10.

**Figure 11:**
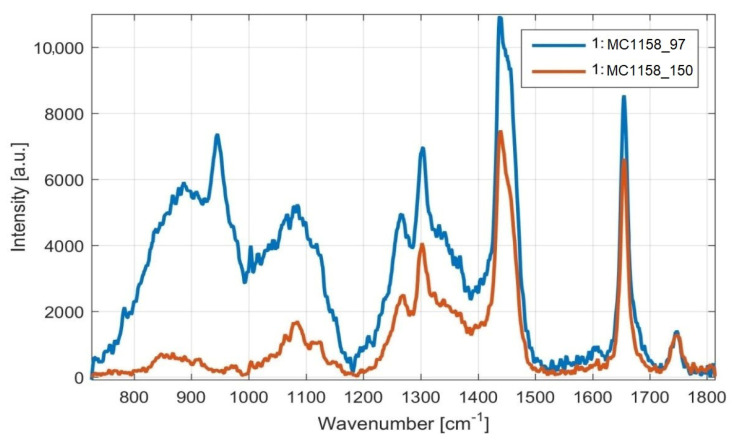
Raman scattering spectrum of intracellular lipids of the yeast *M. chrysoperlae* MC 1158 on two different media. Legend: *Metschnikowia chrysoperlae* 1158 cultivated on medium with C/N ratio 97 (MC1158_97, blue trace) and on medium with C/N ratio 150 (MC1158_150, red trace).

**Figure 12:**
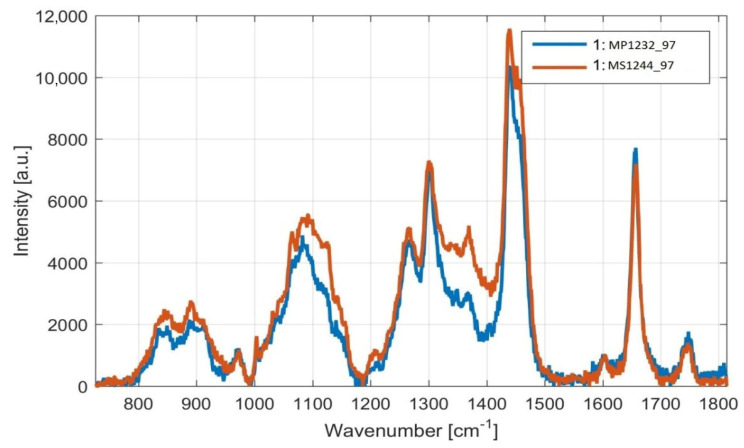
Raman scattering spectrum of intracellular lipids of the yeasts *M. sinensis* and *M. pulcherrima* on a medium of the same composition. Legend: *Metschnikowia pulcherrima* 1232 cultivated on medium with C/N ratio 97 (MP1232_97, blue trace) and *Metschnikowia sinensis* (MS1244_97, red trace).

Detection of fatty acid content by iodine number, using Raman spectroscopy, was demonstrated in selected yeast strains. The results obtained by Raman spectrometry roughly correspond to the results obtained by gas chromatography, but with slight deviations (Figure 11 and Figure 12). These results therefore support the use of Raman spectroscopy in the analysis of microbial lipids, as this method provides a fast and relatively accurate estimate of the FA content with minimal sample modification, which also allows the detection of lipids over time.

As it can be seen from Figure 11 and Figure 12, the ratios of the Raman spectral peaks νU and νS differ from one species to another. *M. chrysoperlae* strain 1158 contained a similar ratio of unsaturated carbon bonds to saturated carbon bonds on the C/N medium at a ratio of 97, as well as at a C/N ratio of 150. The iodine number therefore appeared very similar. GC analysis shows that the amounts of SFA, MUFA and PUFA are indeed very similar (Figure 9). In the second case, in the strain of *M. sinensis* 1244, a higher proportion of saturated carbon bonds was observed over the yeast *M. pulcherrima* 1232. By calculating from the Raman spectra, we obtained a lower iodine number, indicating the presence of a higher proportion of saturated fatty acids. This assumption was confirmed by gas chromatography analysis (Figure 9 and Figure 10).

Raman spectroscopy is a method capable of detecting lipids in cells relatively quickly, reliably and non-destructively. By calculating the ratio of the content of saturated and unsaturated C-C bonds, the iodine number was predetermined for the selected samples, from Raman spectroscopy, which determines the quality of fats and oils.

Although the highest lipid yield in this experiment (19.6%) can be considered relatively significant, some studies have demonstrated the ability of *Metschnikowia* yeasts to achieve higher lipid yields, as compared to the results of this work [23,24,25,26,32]. For this reason, it is necessary to carry out further experiments that would lead to higher lipid production yields, by regulating the cultivation conditions. Raman spectroscopy—a fast and effective tool for the detection of lipid production—can be used to monitor the efficiency of applied nutritional and physical stress conditions.

The fatty acid profiles in lipids produced by yeasts of the genus *Metschnikowia* in this work are comparable to the results of a study [37] where C16 and C18 fatty acids formed majority of the produced saturated fatty acids. However, in the present work, a higher amount of linoleic acid was achieved, which was probably influenced by the change in the ratio of carbon and nitrogen in the medium, while in previously referred-to studies, these conditions were not manipulated [37,38]. From the obtained results, the potential of yeasts of the genus *Metschnikowia* in microbial lipid production is obvious. In addition, it has been shown that, by suitable regulation of cultivation conditions, lipids containing valuable polyunsaturated acids can be obtained, which could have considerable potential in the food and pharmaceutical industry.

## 4. Conclusions

Raman spectroscopy, one of the methods of vibrational molecular spectroscopy, is a technique based on the inelastic scattering of monochromatic radiation known as Raman scattering. As an additional method to infrared spectrometry, it finds application especially in the identification of substances and the determination of their structure and composition. Raman spectroscopy is also commonly used in various sciences in the analysis of solids, liquids and gases, or in the analysis of surfaces or biological systems. The main advantage of this technique is the ability to easily and quickly analyze the sample, without problems with its processing [9,10,39].

Analysis of pulcherrimin production in *Metschnikowia* yeast is very fast and efficient when using Raman spectroscopy. The pigment pulcherrimin is very difficult to isolate, and its presence in cells or media has, in the past, been confirmed mainly by infrared spectroscopy [18]. Raman spectroscopy is a fast and effective tool for the detection of lipid production, too. It can be used to monitor the efficiency of applied nutritional and physical stress conditions.

Raman spectroscopy provides also a quick overview of the saturation or unsaturation of lipids contained in yeast cells. Based on the ratio of specific peaks of unsaturated fatty acids and saturated fatty acids, for example, an increase in unsaturated FA can be observed at the expense of saturated with increasing cultivation time. In addition, to display the time changes of saturated and unsaturated FA detected by Raman spectroscopy, it is also possible to use spectral maps, which very clearly plot this course. In addition, the detection of fatty acids by the iodine-number method showed that the results obtained by Raman spectrometry approximately corresponded to the results obtained by gas chromatography, with slight deviations. 

The presented results of the present study support the use of Raman spectroscopy for rapid monitoring of metabolite production changes during the biotechnological process with minimal sample modification, which also allows the detection of production activity over time. Consequently, the yeasts of the genus *Metschnikowia* can be effectively used for pulcherrimin and/or lipid production, depending on particular stress conditions and media composition.

## Figures and Tables

**Table 1:** Monitoring changes of fatty acids during cultivation in selected strain by gas chromatography (GC). Fatty acid profile (%) of lipid content produced by *Metschnikowia pulcherrima* 149 with C/N ratio 97.

Fatty Acid		Day of Cultivation	
	4	8	14
C15:0	-	-	0.14
C16:0	14.1	13.26	10
C16:1n7	4.45	4.51	4.69
C18:0	12.4	11.48	6.4
C18:1n9	45.51	43.97	39
C18:2n6	19.07	22.73	31.93
C18:3n6	-	0.06	0.14
C18:3n3	0.91	1.28	2.65
SFA *	27.43	25.34	18.66
MUFA *	53	50.48	46.55
PUFA *	19.98	24.17	34.8

* % content of total lipids: SFA—saturated fatty acids, MUFA—monounsaturated fatty acids, PUFA—polyunsaturated fatty acids.

**Table 2:** Effect of the C/N ratio at low temperature on the biomass and lipid production

Strain	C/N Ratio	Biomass (g/L)	Lipid Content (%)
*M. pulcherrima* 149	24	10.4 ± 0.6	5.6 ± 0.5
	97	7.4 ± 0.5	4.4 ± 0.6
	150	7.2 ± 0.5	8.4 ± 0.4
*M. andauensis* 129	24	12.4 ± 0.5	2.2 ± 0.2
	97	6.6 ± 0.5	4.0 ± 0.3
	150	6.2 ± 0.4	3.5 ± 0.2
*M. chrysoperlae* 1158	97	6.9 ± 0.7	12.3 ± 2.4
	150	7.1 ± 0.5	13.6 ± 0.9
*M. pulcherrima* 1232	97	7.0 ± 0.5	14.6 ± 1.9
	150	6.5 ± 0.4	15.4 ± 1.0
*M. sinensis* 1244	97	10.3 ± 1.2	14.4 ± 2.5
	150	9.9 ± 0.6	16.9 ± 1.5
*M. fructicola* 1235	97	15.2 ± 1.4	2.7 ± 0.4
	150	5.6 ± 0.6	4.9 ± 1.3
*M. andauensis* 1241	97	9.3 ± 0.8	17.0 ± 2.2
	150	8.2 ± 0.5	19.6 ± 2.5
